# Alternation of Defects and Phase Turbulence Induces Extreme Events in an Extended Microcavity Laser

**DOI:** 10.3390/e20100789

**Published:** 2018-10-15

**Authors:** Sylvain Barbay, Saliya Coulibaly, Marcel G. Clerc

**Affiliations:** 1 Centre de Nanosciences et de Nanotechnologies, CNRS, Université Paris-Sud, Université Paris-Saclay, Avenue de la Vauve, 91120 Palaiseau, France; 2 Université de Lille, CNRS, UMR 8523-PhLAM—Physique des Lasers Atomes et Molécules, F-59000 Lille, France; 3 Departamento de Física and Millennium Institute for Research in Optics, FCFM, Universidad de Chile, Casilla 487-3, 8370456 Santiago, Chile

**Keywords:** complex dynamics, microcavity laser, spatiotemporal chaos

## Abstract

Out-of-equilibrium systems exhibit complex spatiotemporal behaviors when they present a secondary bifurcation to an oscillatory instability. Here, we investigate the complex dynamics shown by a pulsing regime in an extended, one-dimensional semiconductor microcavity laser whose cavity is composed by integrated gain and saturable absorber media. This system is known to give rise experimentally and theoretically to extreme events characterized by rare and high amplitude optical pulses following the onset of spatiotemporal chaos. Based on a theoretical model, we reveal a dynamical behavior characterized by the chaotic alternation of phase and amplitude turbulence. The highest amplitude pulses, i.e., the extreme events, are observed in the phase turbulence zones. This chaotic alternation behavior between different turbulent regimes is at contrast to what is usually observed in a generic amplitude equation model such as the Ginzburg–Landau model. Hence, these regimes provide some insight into the poorly known properties of the complex spatiotemporal dynamics exhibited by secondary instabilities of an Andronov–Hopf bifurcation.

## 1. Introduction

Out-of-equilibrium systems exhibit permanent complex dynamical behaviors as a consequence of the balance between the injection and dissipation of energy, momentum, and particles [1,2,3]. In particular, nonequilibrium processes often lead in nature to the formation of patterns—dissipative structures [1]—developed from a uniform state thanks to the spontaneous breaking of symmetries present in the system under study [1,2,3,4,5]. Close to this spatial instability, one generically observes the emergence of spatial structures such as stripes and hexagons. As one increases the strength of the control parameter, these patterns exhibit bifurcations that, for example, generate the emergence of more complex stationary patterns such as superlattice and quasi-crystals [5]. One strategy that has allowed a unified description of all these bifurcations and the dynamics of these stationary patterns is based on the amplitude or envelope equations [5,6,7]. As the stationary patterns develop more complex textures, these are described analytically by the inclusion of additional critical amplitudes.

The previous scenario changes radically when the patterns exhibit an oscillatory instability [8], that is, an Andronov–Hopf bifurcation between a stationary pattern to one of an oscillatory nature. The oscillatory patterns are characterized by oscillations in a synchronized manner over a wide range of parameters. By increasing the control parameter, they exhibit a quasi-periodic behavior through a secondary instability [9,10,11]. As a consequence, the Fourier transform of the amplitude shows multiple peaks with incommensurate frequencies. As the control parameter is further increased, this quasi-periodic behavior is replaced by spatiotemporal chaotic behavior. The previous route is known as extended quasi-periodicity [9]. Hence, the pattern exhibits a complex spatiotemporal behavior characterized by a continuous Lyapunov spectrum. Indeed, small modifications or disturbances in the initial conditions generate unpredictability. A simple physical system that presents the former scenario is an extended semiconductor microcavity laser with saturable gain and absorber layers [10,12]. In this system, it has been shown theoretically that spatiotemporal chaos emerges through the mechanism of quasiperiodic, extended spatiotemporal intermittency [10]. The onset of spatiotemporal chaos also gives rise almost simultaneously to extreme events in the form of rare and high amplitude optical pulses. A straightforward correspondence between the proportion of extreme events and the dimension of the strange attractor was established in [12] by comparing experimental and numerical results. The universal envelope model, the Ginzburg–Landau equation [13], which generically describes the dynamics close to an Andronov–Hopf bifurcation, does not adequately account for the dynamics previously described, even though this equation exhibits complex and appealing behaviors such as phase turbulence, amplitude turbulence, and spatiotemporal intermittency [13,14]. Phase turbulence is characterized by a complex dynamics of modes described by a field phase that exhibits a decaying power law in its power spectrum [15]. The corresponding dynamics is of spatiotemporal chaos-type, in which the magnitude of the field is never zero, that is, the real and imaginary parts of the field never cross the zero axis simultaneously. Hence, the field is said to be free of phase singularity or defects in its magnitude. Amplitude turbulence is also characterized by a complex dynamics of modes that exhibit a power law in the field energy power spectrum. However, its main feature is the permanent nucleation of amplitude defects, where the phase is undeterminate [14]. This dynamics requires a strong coupling between the phase and the module of the field envelope. Hence, amplitude turbulence exhibits a dynamical behavior of greater complexity than phase turbulence. The aperiodic emergence of phase singularities characterizes spatiotemporal intermittence, but unlike the dynamics observed in amplitude turbulence, the disappearance of defects is governed by self-organization that engenders transitions between coherent and incoherent regions in the spatiotemporal evolution [14]. Despite the rich dynamics contained in the Ginzburg–Landau equation, this model fails in the adequate physical description of the microcavity laser due to the assumption that the envelope is a slow spatiotemporal variable compared to the wavelength of the underlying pattern. As a consequence of this type of scale mismatch, amplitude equations do not describe several physical phenomena, such as the pinning effect of fronts [16], noise-induced front propagation [17], and the homoclinic snaking bifurcation of localized patterns [18,19].

The characterization of the complex spatiotemporal dynamics exhibited by secondary instabilities of an Andronov–Hopf bifurcation is an open problem in nonlinear science. This paper aims to investigate the complex dynamics shown by the patterns in an extended, one-dimensional semiconductor microcavity laser with an intracavity saturable absorber that displays such secondary instability. Based on a theoretical model, we reveal a dynamic behavior characterized by the chaotic alternation of phase and amplitude turbulence. We stress that this type of dynamics is not contained in the Ginzburg–Landau equation. Interaction and superposition between wave packets characterize phase and defect turbulence [14]. Phase turbulence is distinguished by exhibiting a cascade of the power law for energy versus wavenumber [15]. In the case of defects turbulence, it is characterized by the wave interaction, which permanently gives rise to phase singularities [14]. In the following, we identify the different turbulent behaviors and give new insights into the physical origin of extreme events in our system. Moreover, we find that extreme events occur during the phase turbulence zones.

The manuscript is organized as follows: In Section 2, we review the emergence of extreme events and spatiotemporal chaos in a spatially extended microcavity laser with saturable gain and absorption media. The theoretical model that describes the laser microcavity is presented and analyzed in Section 3. Section 2 and Section 3 constitute a review of our previous results [10,12]. Alternation of defects and phase turbulence in an extended microcavity laser is analyzed in Section 4. Section 5 shows how the alternation of defects and phase turbulence induces extreme events. Finally, conclusions are presented in Section 6.

## 2. Extreme Events in a Microcavity Laser

**Figure 1:**
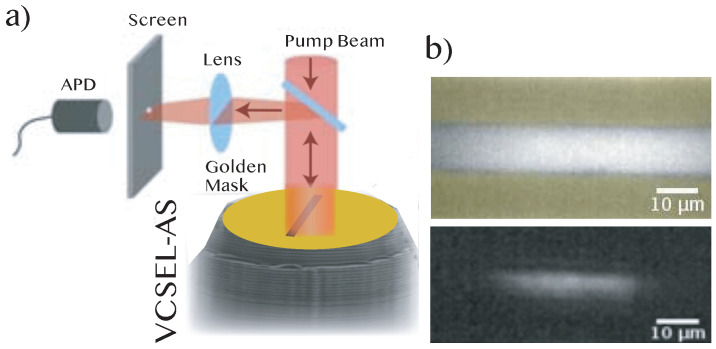
Experimental set up. (**a**) Schematic representation of an extended planar vertical cavity surface emitting laser with an integrated saturable absorber medium (VCSEL-SA). (**b**) Right panels account for the top-view camera snapshots of the one-dimensional line VCSEL-SA surface below (upper image, with the mask visible) and above laser threshold (lower image).

Extreme events have attracted a great deal of attention lately, in particular in optical systems where reliable statistics can be obtained and where many different and controlled physical situations can be explored [20,21]. In dissipative optical systems, extreme events have been found in the intensity dynamics of fibre lasers [22], semiconductor lasers with injected signal [23], and solid-state lasers with a saturable absorber [24]. Vertical-cavity surface emitting lasers with an integrated saturable absorber (VCSEL-SAs) [25,26] are good candidates for studying complex dynamical phenomena and extreme events in self-pulsing spatially extended systems thanks to their small footprint and high aspect ratio. Moreover, the fast timescales associated to semiconductor materials allow for gathering a large amount of information in a short amount of time, which is interesting for statistical analyses and tracking rare phenomena such as extreme events. Broad-area VCSEL-SAs may also have interesting applications, e.g., high-power lasers with vertical cavity emission. These laser devices are composed of two multilayer mirrors, which optimize optical pumping, and of an active zone. This active zone is made up of two InGaAs quantum wells for the gain section and one InGaAs quantum well for the saturable absorber section, forming a 2λ optical length cavity (λ=980 nm). By contrast to a standard laser composed solely of a gain section, the laser with a saturable absorber can sustain self-pulsing at the laser threshold [25]. In the limit of a single transverse mode laser (i.e., with a low aspect ratio cavity), the dynamics is always regular with typical experimental parameters [27]. However, in an extended cavity laser, a more complex dynamics can set in thanks to the interplay between the system nonlinearity and spatial coupling through the light diffraction inside the cavity. In addition, while the typical timescale for the intracavity electromagnetic field is of the order of several picoseconds, the material excitation timescale is much longer (typically the non-radiative recombination of semiconductor carriers is of the order of 1 ns or less). It is thus not possible to reduce the dynamics to the one of the optical intensity. The experimental setup is shown in Figure 1a. The microcavity laser is coated with a thin gold layer with a rectangular opening to define the pumped region. The rectangular mask has an 80 μm length and a 10 μm width, thus forming a quasi one-dimensional line laser. The microcavity laser is optically pumped through a dichroic mirror at 800 nm and emits around 980 nm. Laser emission is imaged on a screen provided with one or two holes. These holes allow for selecting the detection area, which correspond to a disk of a 5 μm diameter on the sample surface. The line VCSEL-SA emission intensity is monitored and recorded with a fast avalanche photodiode (>5 GHz bandwidth). Likewise, the temporal signal is amplified in a low noise, high bandwidth amplifier (3 kHz–18 GHz bandwidth) and acquired with a 6 GHz bandwidth oscilloscope at a sampling rate of 20 GS/s. This allows for easy statistical analysis of the recorded data since very large time traces can be collected in a short amount of time. Figure 1b shows the near field of the laser above threshold with a camera placed at the screen position.

**Figure 2:**
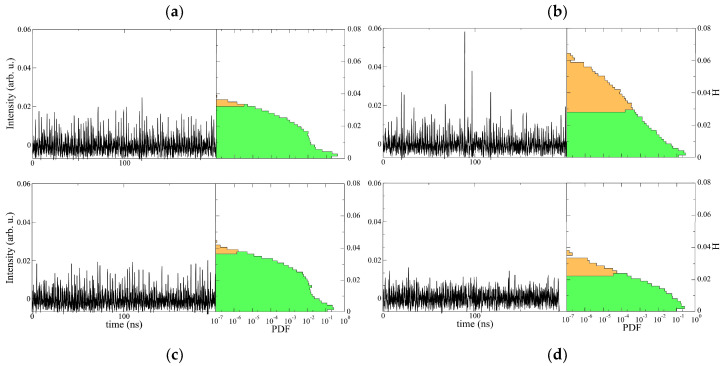
Typical temporal evolution of the experimentally recorded intensity and semi-log graph of the associated probability density distribution of the intensity height *H* for different normalized pump values (adapted from [12]): (**a**) $P/P_{th}=1.02$; (**b**) $P/P_{th}=1.17$; (**c**) $P/P_{th}=1.20$; and (**d**) $P/P_{th}=1.25$. Normal and extreme events are shown in orange and green, respectively (AI>2).

Excerpts of time traces of the laser intensity recorded at the center of the laser are shown in Figure 2 for different pumping intensities. With the full time traces recorded, the histogram of the heights *H* can be constructed. The height *H* is defined by the average of the left and right pulse heights, as in hydrodynamics. From these analyses one can conclude that the system exhibits a complex dynamics of extreme events [10,12]. Figure 2 depicts heights histograms for different values of the pump parameter *P*. Let us introduce $P_{th}$ as the laser threshold pump. At normalized pump power $P/P_{th}=1.02$, the histogram in a semi-log plot is characterized by a quadratic decay in the tails. Figure 2a shows the probability density function (PDF), which resembles a Rayleigh distribution for a positive valued Gaussian process. Increasing the pump parameter, the PDF develops long tails with an initial exponential decay (cf. Figure 2b). Increasing further the pump values, the PDF becomes an exponential distribution ($P/P_{th}=1.20$). For a still higher pump value ($P/P_{th}=1.25$) the PDF redisplays a Gaussian tail. To determine the threshold amplitude for extreme events, we consider the standard hydrodynamical criterion, that is, an extreme event corresponds to an event having a height *H* twice the significant height $H_{s}$, where $H_{s}$ stands for the mean of the highest tertile of the PDF. Namely, extreme events are characterized by an abnormality index $AI\equiv H/H_{s}>2$ [28]. To ignore a large number of small peaks due to detector noise to the left of the PDF, one can determine the relevant or significant height $H_{s}$ by considering events whose altitude is higher than the observed maximum peak dark noise amplitude. On Figure 2, extreme events are in orange in the PDF. When the PDF presents a non-Gaussian tail, we observe that the system exhibits a large number of extreme events (a normalized pump of 1.17). When increasing the pump parameter, a complicated dynamical behavior characterized by intermittent pulsations of the recorded intensity is observed. Indeed, the dynamics shows irregular oscillations of the intensity characterized by sharp peaks that appear irregularly in the temporal domain; that is, the peaks exhibit an aperiodic behavior, which is a typical signature of chaos [10]. Hence, the dynamics of the microcavity laser is characterized by a supercritical intermittency route to chaos [29], and has thus been called extended spatiotemporal intermittency [10]. The experimental results discussed so far are well reproduced by a theoretical model of an extended microcavity laser with a saturable absorber, which we present in Section 3.

**Figure 3:**
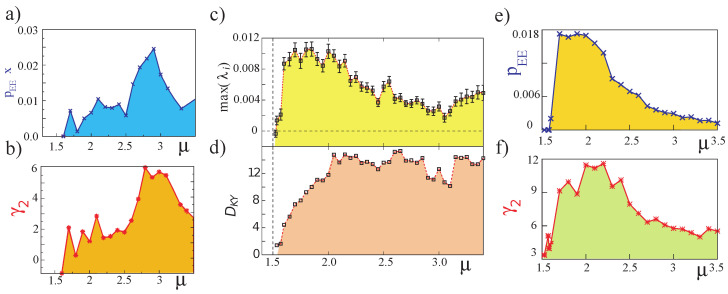
Numerical characterization of the emergence of extreme events in an extended, planar vertical-cavity surface-emitting laser with an integrated saturable absorber medium obtained from Equation (1). Graph of the proportion of extreme events $p_{\mathrm{EE}}$ (×) (**a**) and excess kurtosis $\gamma_{2}$ (∗) (**b**) as a function of pump parameter $\mu =P/P_{th}$ considering the height *H* of the laser intensity. Graph of the largest Lyapunov exponent $\max(\lambda_{i})$ (squares) (**c**) and Kaplan–Yorke dimension $D_{\mathrm{KY}}$ (**d**) as a function of pump parameter μ. Graph of the proportion of extreme events $p_{\mathrm{EE}}$ (**e**) and excess kurtosis $\gamma_{2}$ (**f**) as a function of pump parameter μ considering the local intensity spatiotemporal maxima (adapted from [10]).

The emergence of extreme events is related to the onset of spatiotemporal chaos, or at the beginning of the transition from a complex dynamical behavior to another [10,12]. The total intensity $I_{\mathrm{tot}}\left(t\right)\equiv \int {\left|E\left(x,t\right)\right|}^{2}dx$ and local intensity $I_{\mathrm{loc}}\equiv {\left|E\left(x,t\right)\right|}^{2}$, where E(x,t) is the intracavity electric-field envelope, are two relevant physical quantities to characterize the dynamics of the extended microcavity laser. The latter quantity, in particular, is only accessible through numerics because it is not possible to record the full spatiotemporal evolution in the experiment, due to the very short timescales at stake. This justifies the numerical approach that we present hereafter. Figure 3a,b show the proportion of extreme events in all the numerically observed events ($p_{\mathrm{EE}}$), and the deviations from the Gaussian distribution of the numerical PDF (excess kurtosis $\gamma_{2}$) as a function of the pump parameter μ. The same analysis is done for the two observables, namely the total intensity emitted by the laser $I_{\mathrm{tot}}$ (cf. Figure 3a,b) and the intensity of the spatiotemporal peaks $I_{\mathrm{loc}}$ (cf. Figure 3e,f). Note that $p_{\mathrm{EE}}$ and $\gamma_{2}$ are correlated in both cases. However, they follow different trends with μ: in the case of the observables associated with the intensity, both extreme events indicators tend to grow as a function of the pumping parameter. However, extreme events indicators linked to spatiotemporal intensity peaks tend to increase near the bifurcation of the spatiotemporal chaos and subsequently decay strongly.

## 3. Theoretical Description of a One-Dimensional Spatially Extended Laser

A planar vertical-cavity surface-emitting line laser with a saturable absorber medium can be described to a good approximation by a one-dimensional spatially extended laser with a saturable absorber layer [30]. This model has been shown to successfully describe different phenomena in the system under study, such as pattern and localized structure formation [31] and spatiotemporal chaos. In this latter case, we have shown that the model captures very well the evolution with the pump parameter of the intensity statistics and of the intensity cross-correlation computed at two different locations, as well as the evlution of the power spectrum of the intensity and of the extreme event indicators [10,12]. The dimensionless model reads

(1) $$\begin{array}{ccc} \frac{\partial E(x,t)}{\partial t} & = & \left[(1-i\alpha )G+(1-i\beta )Q-1\right]E+i\frac{\partial^{2}E}{\partial x^{2}}\\ \frac{\partial G(x,t)}{\partial t} & = & \gamma_{g}\left[\mu -G(1+|E|{}^{2})\right]\\ \frac{\partial Q(x,t)}{\partial t} & = & \gamma_{q}\left[-\gamma -Q(1+s|E{|}^{2})\right] \end{array}$$

where the fields E(x,t), G(x,t), and Q(x,t), respectively, account for the intracavity electric-field envelope, the carrier density in the gain, and the saturable absorber medium. *x* and *t* stand for the spatial coordinate and time. The non-radiative carrier recombination rates are $\gamma_{g}$ and $\gamma_{q}$. The parameters μ and γ are the pumping and linear absorption processes, respectively. The parameters α and β account for the Henry enhancement factors in both the gain and absorber regions, respectively. These parameters are related to phase-amplitude coupling in semiconductor media. The Laplacian term stands for the diffraction process. Diffusion processes of carriers are smaller than diffraction ones and are ignored in the first approximation. The time and spatial variables have been rescaled to the field lifetime and the diffraction length $w_{d}$ in the cavity, respectively. Considering the parameters of the cavity, the time and spatial scales correspond to 8.0 ps and 7.4 μm. Since the pumped region has a length $w_{p}∼80\;\mu$m, we obtain $w_{p}/w_{d}∼11$ as a direct estimate of the Fresnel number of the line microlaser. Considering parameters compatible with our semiconductor system, we obtain α=2, β=0, s=10, $\gamma_{g}=\gamma_{q}=0.005$, and γ=0.5. The Henry enhancement factors are chosen with usual values [32]. Assuming that the carriers recombinations times are of the order of 800 ps, one can determine the other physical parameters straighforwardly.

The bifurcation diagram of Equation (1) has been studied in detail (see [30] and references therein). For small pumping, the system is in the no-lasing state. When increasing the pumping parameter above $\mu_{th}=1+\gamma$, the (plane-wave) lasing threshold is reached. Further increasing the pumping parameter, Equation (1) exhibits an Andronov–Hopf bifurcation for plane waves $\mu \left(I\right)<\mu_{H}\left(I\right)\equiv r\left(2rsI\gamma -\gamma_{g}\left(1+I\right)\left(1+sI\right)\left(1+I+r+rsI\right)\right)/2I$ with $r=\gamma_{q}/\gamma_{g}$ [30]. Due to the complex dynamics presented by the system, analytical studies are inaccessible. To figure out the dynamics exhibited by the microcavity extended laser with a saturable absorber medium, we have numerically studied model (1). Our strategy has been to consider only one parameter in the analysis, for better comparison with the experiment where this parameter is easily accessible, namely the power pump parameter μ. For pumping power values such that $\mu >\mu_{th}$, the laser turns on through a transcritical bifurcation. When increasing the pump power value ($\mu /\mu_{th}∼1.047$), the total intensity $I_{\mathrm{tot}}$ exhibits a quasi-periodic dynamical behavior. Indeed, the temporal evolution of the total intensity of the electric field envelope is aperiodic and presents fluctuations around its average value [10]. Note that extreme events are not observed in this parameter regime. Unexpectedly, increasing the value of the pumping power parameter ($\mu /\mu_{th}∼1.333$), the system presents a bifurcation. In this parameter regime, the total intensity exhibits intermittent pulsations in its temporal evolution characterized by aperiodic fluctuations, in which sharp peaks randomly appear. This dynamical behavior is compatible with the experimental observations as shown in Figure 2.

To understand the complex dynamics observed, we can determine its sensitivity to perturbations by means of the Lyapunov spectrum (with Lyapunov exponents $\lambda_{i}$). One of the main characteristics of this spectrum is that the system presents a temporal or low dimensional chaotic behavior if and only if the largest Lyapunov exponent $\max(\lambda_{i})$ is positive. However, to conclude a spatiotemporal or high dimensional chaos, the latter condition is necessary but not sufficient. Spatiotemporal chaos is a permanent, aperiodic spatiotemporal dynamical behavior. In addition, this dynamical behavior is characterized by being of an extensive nature [33]. The Lyapunov spectrum is composed by the set of the Lyapunov exponents arranged in decreasing order considering their real parts. This spectrum allows the distinction between chaos and spatiotemporal chaos. Indeed, a Lyapunov spectrum with a continuous set of positive values characterizes spatiotemporal chaos. In contrast, a Lyapunov spectrum with a discrete set of positive values characterizes chaos of low dimensions. The Kaplan–Yorke dimension $D_{KY}$ [34] can be determined from the Lyapunov spectrum. This dimension accounts for the dimension of the strange attractor under study. The largest Lyapunov exponent $\max(\lambda_{i})$ and the Kaplan–Yorke dimension are right quantities to characterize complex dynamical behaviors and transitions between them [35]. For instance, steady-state solutions are characterized by a negative and zero largest Lyapunov exponent and Kaplan–Yorke dimension, respectively. Periodic or quasi-periodic solutions have a zero largest Lyapunov exponent and Kaplan–Yorke dimension. When both the largest Lyapunov exponent and Kaplan–Yorke dimension are strictly positive, this corresponds to a chaotic dynamical behavior. In the region of aperiodic intermittent pulsations, Equation (1) shows a characteristic Lyapunov spectrum of a spatiotemporal nature [10,12]. Figure 3c,d show $\max(\lambda_{i})$ and $D_{KY}$ as a function of the pumping parameter μ, obtained numerically. We observe that the emergence of extreme events in the microcavity laser is correlated to the appearance of spatiotemporal chaos. Indeed, extreme events are observed only when the largest Lyapunov exponent and the Kaplan–Yorke dimension are both strictly positive.

In addition, when increasing the pump power parameter, the spatiotemporal complexity increases (see the onset of spatiotemporal chaos in Figure 3). Note that $\max(\lambda_{i})$ and $D_{KY}$ both consistently increase with the pumping value μ. The microcavity laser with a saturable absorber medium exhibits extreme events when the system is in a regime of spatiotemporal chaos. However, the kind of spatiotemporal chaos displayed by Equation (1) is not determined by this analysis and will be the subject of the next section.

## 4. Characterization of Spatiotemporal Dynamics of an Extended Laser with a Saturable Absorber: Alternation of Defects and Phase Turbulence

**Figure 4:**
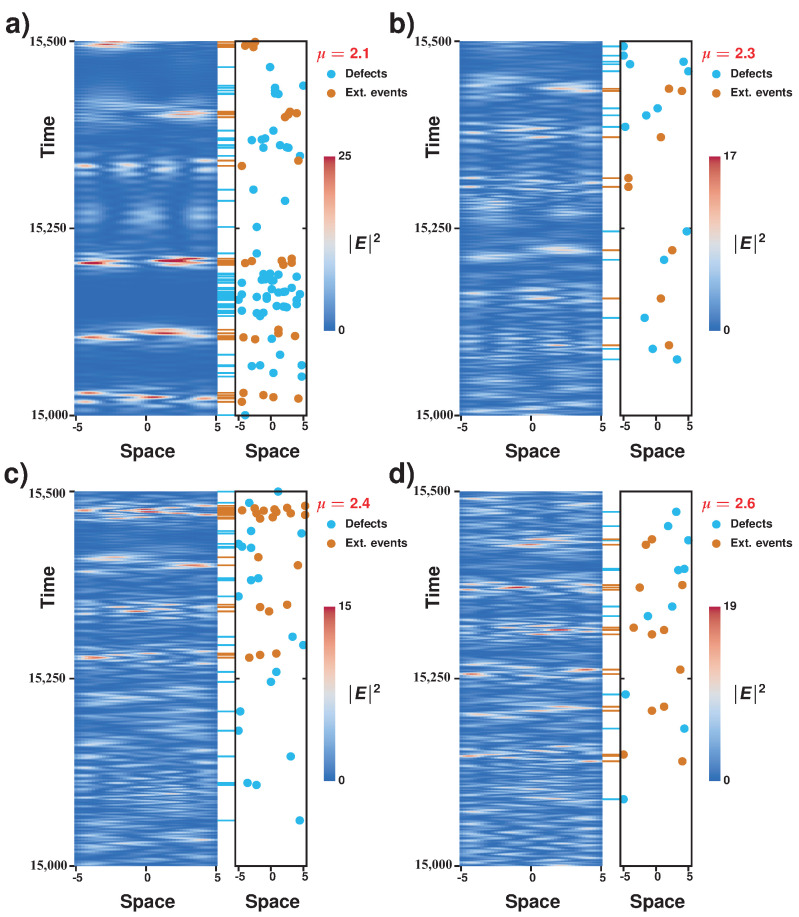
Alternation of defects and phase turbulence in the laser with saturable absorber model expressed by Equation (1). Spatiotemporal evolution of the electric field intensity, together with the spatiotemporal positions of phase singularities of the electric field envelope E(x,t) and of the extreme events (blue and red dots, respectively; temporal location of respective events are highlighted by dash signs) in the spatiotemporal complex regime with α=2, β=0, $\gamma_{g}=0.005$, $\gamma_{q}=0.005$, γ=0.5, s=10, and the following μ values: (**a**) μ=2.1; (**b**) μ=2.3; (**c**) μ=2.4; (**d**) μ=2.6.

To figure out the complicated dynamical behaviors presented by the microcavity laser model with a saturable absorber, we simulated numerically the set of Equation (1). We used a split-operator method to accurately compute the Laplacian term, while the nonlinear temporal evolution is taken care of in real space. The non-zero pump is restricted to a finite domain ([−5,5] interval) and is zero otherwise (not shown), thus giving absorbing boundaries. Figure 4 displays the space–time evolution of the laser intensity together with spatiotemporal positions of defects and of extreme events computed for different pumping parameters. Defects correspond to zeros of the envelope of the electric field E(x,t); that is, in these points, the phase is not defined: they correspond to phase singularities [13]. From this figure, we observe that the system presents interchange between a region of phase turbulence and defects turbulence. The region of phase turbulence is characterized by a complex dynamics of wave interaction. In this region, the phase is always well defined; that is, the amplitude of the waves is never zero. Note that, in this region, the spatial modes of the system exhibit complex spatiotemporal dynamics (cf. Figure 4). We monitored and determined the spatiotemporal positions of the amplitude defects in the temporal progression of the envelope of the electric field (see blue dots in Figure 4). Note that amplitude defects tend to gather for low pumping and generally display a complex spatiotemporal distribution. The regions of phase turbulence are separated by areas with low intensities that exhibit amplitude defects (phase singularities). Likewise, we monitored and determined the spatiotemporal position of extreme events in the electric field envelope *E* (see red dots in Figure 4 and corresponding dash signs). One expects complex behaviors such as phase or defects turbulence to exhibit extreme events due to the strong temporal correlation of deterministic dynamics. Unexpectedly, extreme events are mostly observed in the regions of phase turbulence. We can therefore conclude that the spatiotemporal dynamics of the system is characterized by the chaotic alternation of phase singularities (amplitude defects) and the observation of large amplitude pulsations (extreme events). This type of complex spatiotemporal dynamics is not contained in universal models, such as the Kuramoto–Sivashinsky [15] and the Ginzburg–Landau equations [13], which account for the dynamics around an Andronov–Hopf instability. Hence, the dynamics observed in Equation (1) goes beyond the dynamics contained arround the Andronov–Hopf bifurcation, and the alternation between phase and defects turbulence is a new kind of complex dynamics.

**Figure 5:**
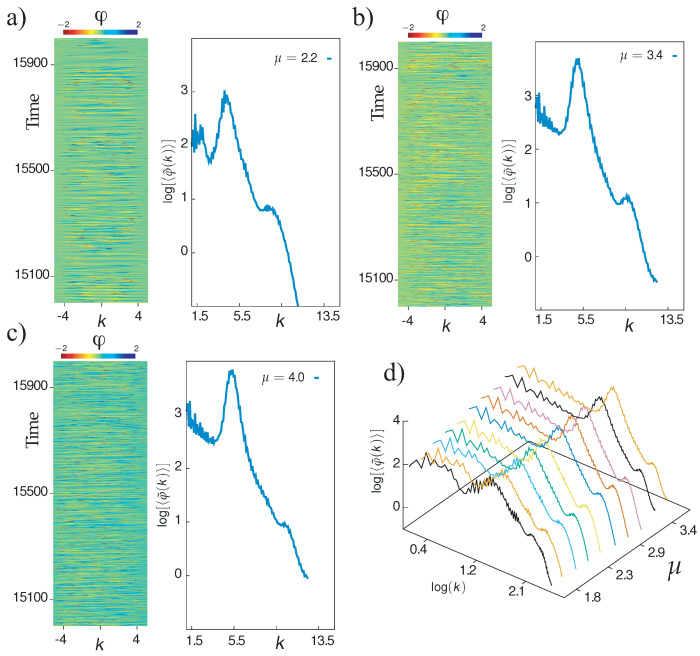
Turbulence dynamics of the one-dimensional microcavity laser with a saturable absorber medium. Spatiotemporal diagram and the average spectrum ${\bar{\varphi }}_{k}$ of the phase of the electric field envelope of Equation (1) by α=2, β=0, $\gamma_{g}=0.005$, $\gamma_{q}=0.005$, γ=0.5, s=10, and the following μ values: (**a**) μ=2.2; (**b**) μ=3.4; (**c**) μ=4.0. (**d**) The average spectrum ${\bar{\varphi }}_{k}$ of the phase of the electric field envelope for different pumping parameters.

To characterize more accurately the dynamics exhibited by the system, we calculate the phase associated with the envelope

(2) $$\varphi \left(x,t\right)\equiv \frac{ℑ[E(x,t)]}{ℜ[E(x,t)]}$$

and analyze its spatiotemporal evolution. Close to the Andronov–Hopf bifurcation, the equations governing the phase and envelope amplitude can be decoupled. Notably, around the Benjamin–Feir instability [2], the phase satisfies the Kuramoto–Sivashinsky equation. This model has been an angular footing in the study of complex spatiotemporal dynamics, since it corresponds to the simplest scalar model that describes the dynamics of coupled oscillators and exhibits turbulence dynamics [15]. Likewise, this is one of the first models to be used to rigorously unveil spatiotemporal chaos and display a continuous Lyapunov spectrum [36]. However, the dynamics displayed in the spatiotemporal diagrams of the amplitude (cf. Figure 4) shows a regular appearance of phase singularities, which is a prohibitive condition for the separation of dynamics from the phase and the magnitude of the envelope. This rules out a mechanism similar to the one found in the Kuramoto–Sivashinsky equation. We investigated the spatiotemporal evolution of the phase as defined by Equation (2) for different values of the pumping parameter and plotted the result in Figure 5. These diagrams illustrate a complex wave dynamics since no visible structure emerge. To characterize this dynamic from a statistical point of view, we calculate the average spectrum of phase fluctuations defined by [15]

(3) $$\langle \bar{\varphi }\left(k\right)\rangle \equiv \lim_{T\to \infty }\frac{1}{T}\int_{0}^{T}{\left[\frac{1}{L}\int_{-L/2}^{L/2}\varphi \left(x,t\right)e^{ikx}dx\right]}^{2}dt$$

*L* accounts for the system size, *T* is a long enough time, to perform an average on the statistics, and *k* is a wavenumber. This quantity allows one to characterize the transport of energy between the different scales of the coupled oscillators [15]. Figure 5 shows the average spectrum of phase fluctuations $\langle \bar{\varphi }\left(k\right)\rangle$ for different pumping values in semi-log and log-log plot. It is clearly visible there that the averaged phase spectrum exhibits a power-law behavior in a specific range of wave numbers. From this observation, one can conclude that the dynamics presented by the microcavity laser with a saturable absorber medium is of a turbulent nature. Hence, the dynamical behavior characterized by alternation of defects and phase spatiotemporal complexity is of a turbulent nature.

## 5. Alternation of Defects and Phase Turbulence Induces Extreme Events

**Figure 6:**
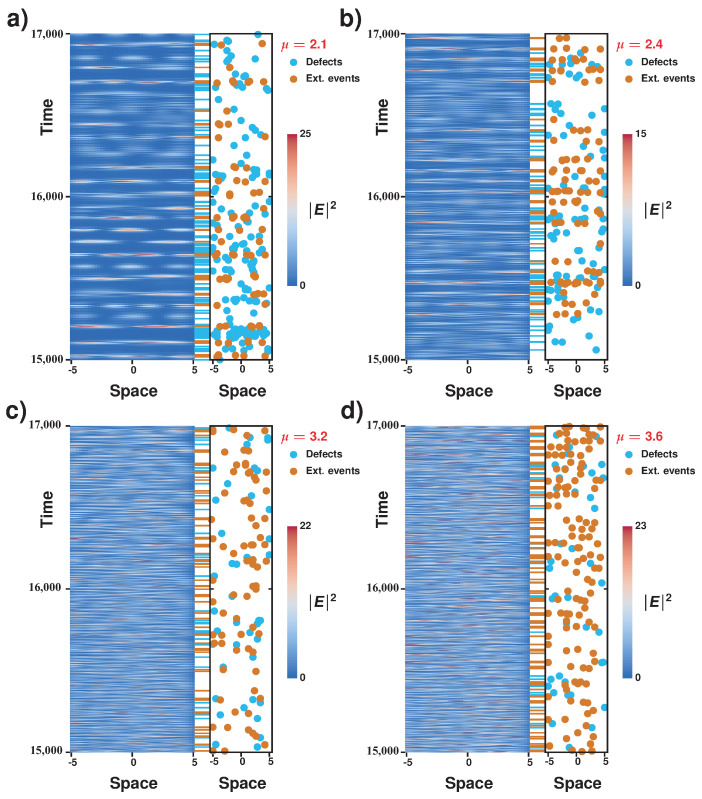
Complex dynamics exhibited by the laser with saturable absorber model computed with Equation (1) in a large time window. Spatiotemporal progression of the electric field magnitude and spatiotemporal positions of the defects of the electric field envelope E(x,t) and of the extreme events (blue and red dots, respectively; temporal location of respective events are highlighted by dash signs). Parameters are identical to those in Figure 4, with pumping: (**a**) μ=2.1; (**b**) μ=2.4; (**c**) μ=3.2; and (**d**) μ=3.6.

In order to emphasize the relationship between the alternation dynamics from phase turbulence to defects turbulence and the appearance of extreme events, we analyzed the spatiotemporal diagrams in a larger simulation time window in Figure 6, and for different pumping parameters. Near the lasing bifurcation, there are globally many defects and those have a tendency to bunch in the low laser intensity zones to give clear alternations with the zones of phase turbulence where, by contrast, extremes events can be found. The chaotic pulsation (and the alternation dynamics between the turbulent regimes) consists of large areas of defect turbulence (low intensity zones) and small areas of phase turbulence (higher intensities), which in turn is consistent with the observation of a large number of extreme events (i.e., rare and high intensity peaks). However, as one moves away from the bifurcation point, the number of defects is much smaller and amplitude defects tend to spread all over the spatiotemporal diagram. This is consistent with a faster alternation of the turbulent regimes (defects and phase mediated) and with the fact that the proportion of extreme events globally decreases (see Figure 3).

As illustrated in Figure 3, extreme events appear almost simultaneously with the emergence of spatiotemporal chaos. One can understand this phenomenon because the observed spatiotemporal chaotic dynamics is of an intermittent nature, namely, the system moves between different dynamical behaviors. However, the occurrence of spatiotemporal chaos does not necessarily mean in general that the system will display extreme events. Nonetheless, the aperiodic alternation between different (complex) dynamical behaviors can generate extreme events. That is, chaotic behaviors that are characterized by the variation between different dynamical behaviors is a natural context where one can observe extreme events. The above argument explains why in the laser with a saturable absorber, one verifies the simultaneous emergence of extreme events and spatiotemporal chaos. It can also allow one to establish a parallel with the context of (temporal) chaos, where it has been shown that deterministic extreme events are linked to multistability and to the occurrence of crises [37,38].

## 6. Conclusions

Out-of-equilibrium extended systems exhibit complex dynamical spatiotemporal behaviors. One strategy for understanding this type of dynamical behavior is to investigate its bifurcations and routes to complexity. Nevertheless, the greatest successes have been achieved in understanding primary instabilities, thanks to the use of amplitude equations, perturbation singular, and normal forms theory. The characterization and classification of complex behaviors in extended systems are one of the fundamental problems of nonlinear science. We investigated the complex dynamics shown by oscillatory patterns in a spatially extended semiconductor microcavity laser with an intracavity saturable absorber. Based on a theoretical model of the microcavity laser, which has proven to be qualitatively accurate in the experimental system’s description, a numerical analysis has revealed a complex spatiotemporal dynamical behavior characterized by the alternation of phase and amplitude turbulence. To our knowledge, this is the first time that this intriguing dynamical behavior has been reported since the two turbulent regimes are usually not observed in current models within the same parameter regions. It is also remarkable to note that this kind of dynamics is beyond the Ginzburg–Landau world [13]. Likewise, the alternation between turbulent behaviors is characterized by the occurrence of the highest amplitude optical pulses, which are observed in the phase turbulence zones. Indeed, it was already known that the appearance of spatiotemporal chaos generates extreme events, but we give here a much finer account of the kind of dynamical mechanism that is responsible for the observation of extremes. At last, the complex spatiotemporal dynamics observed here is believed to be observable in other systems that exhibit an Andronov–Hopf bifurcation. Work in this direction is in progress.
